# Comparing Approaches for Estimating Counterfactual HIV Incidence Among Populations With High Vulnerability to HIV in Lima, Peru: A Multi‐Study Comparative Analysis

**DOI:** 10.1002/jia2.70204

**Published:** 2026-09-15

**Authors:** Fei Gao, Sayan Dasgupta, Siavash Pasalar, Qi Wang, Ricardo Alfaro, Delia Pinto‐Santini, Thiago S. Torres, Jorge Sanchez, Javier R. Lama, Valdilea G. Veloso, Michal Juraska, Robinson Cabello, Jorge O. Alarcon, Beatriz Grinsztejn, Carlos F. Caceres, James F. Rooney, Ann Duerr

**Affiliations:** ^1^ Vaccine and Infectious Disease Division Fred Hutchinson Cancer Research Center Seattle Washington USA; ^2^ Public Health Sciences Division Fred Hutchinson Cancer Research Center Seattle Washington USA; ^3^ Department of Biostatistics and Bioinformatics Duke University Durham North Carolina USA; ^4^ Centro de Investigaciones Tecnológicas Biomédicas y Medioambientales (CITBM) Lima Perú; ^5^ Fundação Oswaldo Cruz Instituto Nacional de Infectologia Evandro Chagas Rio de Janeiro Brazil; ^6^ Asociación Civil Impacta Salud y Educación Lima Perú; ^7^ Asociación Vía Libre Lima Peru; ^8^ Universidad Nacional Mayor De San Marcos Lima Peru; ^9^ Universidad Peruana Cayetano Heredia Lima Peru; ^10^ Gilead Sciences Foster City California USA

**Keywords:** avidity, gonorrhoea, HIV pre‐exposure prophylaxis, human immunodeficiency virus (HIV), longitudinal cohort, propensity score weighting

## Abstract

**Introduction:**

The availability of highly efficacious HIV pre‐exposure prophylaxis (PrEP) makes it unethical or infeasible to conduct inactive/placebo‐controlled trials to evaluate new HIV prevention options. As a result, randomized active‐control non‐inferiority trials are typically employed; however, they require large sample sizes and extended follow‐up. Moreover, their results can be hard to interpret. An alternative approach compares HIV incidence among individuals receiving a new PrEP product to a counterfactual incidence estimate—an estimate of what the HIV incidence would have been in the absence of PrEP.

**Methods:**

We leveraged data from three studies (AMP, Sabes and ImPrEP seroincidence) conducted among men who have sex with men and transgender persons in Lima, Peru, between 2013 and 2022 to estimate the counterfactual HIV incidence for two target populations represented by a clinical trial (AMP) and a non‐interventional cohort study (Sabes). We evaluated three estimation methods: prospective cohort follow‐up, recency testing and rectal gonorrhoea (RG) approaches. These approaches were compared directly using data from the same study, and we further assessed population adjustment approaches by comparing estimates across studies.

**Results:**

All three methods produced consistent HIV incidence estimates when applied to data collected from the same study. ​However, estimates differed when data from external studies were used, even after propensity score (PS) adjustment. For example, estimates for the AMP population using Sabes or ImPrEP data remained higher than the AMP gold‐standard estimate. In contrast, adjusted estimates for the Sabes population using AMP or ImPrEP data were lower than the Sabes follow‐up estimate. These differences underscore the challenges of applying external data and highlight the role of unmeasured population heterogeneity.

**Conclusions:**

Counterfactual HIV incidence estimates can support evaluation of new PrEP agents when direct placebo comparisons are not possible. Estimates from recency‐testing and RG approaches are promising when prospective follow‐up is infeasible, but their validity depends on data quality and population similarity. PS methods improve comparability across populations but cannot fully account for unobserved differences. Triangulating across methods and sources can improve confidence in counterfactual estimates, which should be interpreted with careful attention to population context.

## 1 Introduction

Highly efficacious HIV pre‐exposure prophylaxis (PrEP) agents have been developed over the past decade. Daily oral emtricitabine/tenofovir disoproxil fumarate (FTC/TDF) reduces HIV risk by over 90% when taken as prescribed [1, 2, 3], and has also shown high effectiveness in an event‐driven or “on‐demand” regimen among men who have sex with men (MSM) [4]. Injectable cabotegravir (CAB‐LA) has demonstrated even greater efficacy among MSM and transgender persons (TG) [5, 6]. More recently, twice‐yearly lenacapavir has shown 100% and 96% efficacy in HIV prevention among cisgender women [7] and men and gender‐diverse persons [8], respectively.

The high efficacy of existing PrEP agents poses challenges for evaluating new HIV prevention options. Incorporating the current standard of care makes placebo‐controlled trials unethical or infeasible. As a result, a randomized active‐control non‐inferiority design is typically used. This design requires a larger sample size and longer follow‐up than placebo‐controlled designs [9], and because both arms are expected to have low incidence, interpreting results can be challenging due to the small number of HIV acquisitions.

Challenges in implementing traditional clinical trial designs for evaluating new HIV prevention agents have led to the development of alternative approaches. One such approach compares HIV incidence among individuals receiving an experimental PrEP product to a counterfactual incidence estimate—an estimate of what the incidence would have been without PrEP. Various methods have been proposed to estimate this counterfactual, including use of HIV follow‐up data from placebo arms of concurrent trials [10] and cross‐sectional recency testing data collected among screened participants to detect recent acquisitions among individuals living with HIV [9, 11, 12], as applied in the PURPOSE 1 and 2 trials [7, 8]. Additional strategies include leveraging prospective follow‐up data on HIV acquisitions associated with similar risk behaviours, such as rectal gonorrhoea (RG) [13], community HIV surveillance data [14] and adherence data from participants assigned to daily oral F/TDF [15, 16].

There are few, if any, publications that compare different methods for estimating the counterfactual HIV incidence—either against one another or against the gold standard of prospectively measured HIV incidence among individuals not taking PrEP. Such comparisons are inherently challenging, as estimates based on concurrent data are preferred [11] due to changes in HIV incidence and population characteristics. However, it is rare to have data for multiple methods collected during the same time period and in the same geographic area, limiting the feasibility of direct comparison.

In this paper, we directly compare approaches for estimating counterfactual HIV incidence, leveraging a unique setting in Lima, Peru, where multiple well‐controlled studies among MSM and TG were conducted within a relatively short time frame. Lima has a concentrated HIV epidemic among sexual and gender minorities, reflecting a high regional burden [17, 18]. We examine three approaches: (1) a prospective follow‐up approach using concurrent cohort data; (2) a recent‐testing approach using screening data; and (3) an RG approach using prospective RG data. These methods are applied to data collected from three studies conducted in Lima between 2013 and 2022, enabling a rare, head‐to‐head comparison within a shared geographic and epidemiologic context.

## 2 Methods

### 2.1 Data

We used data from three studies conducted among MSM and TG in Lima, Peru between 2013 and 2022, with key features summarized in Table 1. Approval for this analysis was obtained from the Fred Hutch IRB; all participants in the three studies had provided written informed consent, including consent for future use of data.

- **Antibody‐Mediated Prevention (AMP) trial (HVTN 704/HPTN 085)**. This randomized trial enrolled 2699 HIV‐negative MSM and TG across 26 sites in Brazil, Peru, Switzerland and the United States between April 2016 and October 2018 [19, 20]. Participants were randomized in a 1:1:1 ratio to receive placebo, low‐dose or high‐dose VRC01, a monoclonal antibody. HIV testing was conducted every 4 weeks for the first 80 weeks, and every 8 weeks thereafter through 104 weeks, with follow‐up completed in April 2020. Since the estimated HIV incidence did not differ significantly between arms, we included data on HIV and RG incidence from all arms for the 886 participants enrolled and followed at four Lima sites (Barranco, San Miguel, Via Libre and CITBM).
- **Sabes study**. This study investigated the feasibility and benefits of incorporating frequent testing for acute and recent HIV acquisitions and rapid linkage to care at community testing and treatment sites among MSM and TG in Lima [21]. In step 1, 3337 participants with high vulnerability to HIV were screened between July 2013 and September 2015 across four Lima sites (Barranco, San Miguel, Via Libre and Epicentro). These included 228 participants from the 2011 Peruvian Biobehavioral Survey, initially tested for HIV and confirmed HIV‐negative. As they differed from the general screening population, they were excluded from our analysis, resulting in a total of 3109 participants. Among these, 629 participants received an HIV‐positive test result at the screening visit, and 614 had blood samples evaluated with a recent infection testing algorithm (RITA) that includes limiting antigen avidity (LAg‐Avidity) (Maxim [22]) and viral load. Among the participants who tested HIV‐negative, 1912 were enrolled in step 2 and followed with monthly HIV testing for up to 2 years (follow‐up completed in May 2016). PrEP was not available in Peru during the study period.
- **ImPrEP seroincidence study**. This cross‐sectional study enrolled sexual and gender minorities not using PrEP or post‐exposure prophylaxis (PEP) from 18 large urban centres in Brazil and Peru [18]. Participants were recruited from HIV counselling and testing units, sexually transmitted infection clinics and HIV prevention services within the public health system. The study aimed to identify factors associated with recent HIV acquisitions, estimate annualized HIV incidence and calculate the number of people with averted HIV acquisitions during the oral PrEP implementation study [23]. Between January 2021 and May 2022, 6899 individuals were tested for HIV. We included the 1029 participants screened at five sites in the metropolitan area of Lima (Centro de Salud Gustavo Lanata, Centro de Salud Madre Teresa de Calcuta, Centro Materno Infantil San José, Centro Materno Infantil Tahuantinsuyo Bajo, Centro de Salud Alberto Barton), of whom 160 received an HIV‐positive test result and completed recency testing (LAg‐Avidity, viral load and CD4).

**TABLE 1:** Overview of studies included for counterfactual HIV incidence estimation in Lima, Peru.

Study	Study period	Main eligibility criteria	HIV prevention product use	HIV follow‐up incidence	Recency testing at enrolment	RG incidence
AMP	04/2016−04/2020	Age 18–50; born male or identifying as TG; at high risk of acquiring HIV	Randomized to placebo, low‐dose or high‐dose of VRC01; offered HIV prevention package including oral PrEP	Yes	No	Yes
Sabes	07/2013−05/2016	Age 18+; male at birth; had sex with a male in the past 12 months; unaware of HIV status; at high risk of acquiring HIV	None	Yes (step 2)	Yes (step 1)	No
ImPrEP seroincidence	01/2021−05/2022	Age 18+; male at birth; self‐identify as MSM or TG; not using PrEP or PEP	None	No	Yes	No

Abbreviations: AMP, Antibody‐Mediated Prevention trial; MSM, men who have sex with men; PEP, post‐exposure prophylaxis; PrEP, pre‐exposure prophylaxis; RG, rectal gonorrhoea; TG, transgender persons; VRC01, VRC01 broadly neutralizing monoclonal antibody.

Although these studies were conducted within a shared geographic setting, they spanned different calendar periods between 2013 and 2022 and enrolled populations with potentially different risk profiles and prevention access. During this period, HIV prevention availability, testing practices and engagement in care changed in Peru, and these temporal differences may have contributed to variation in HIV incidence across studies. Therefore, our primary goal was not to demonstrate identical HIV incidence estimates across studies, but rather to evaluate whether different counterfactual estimation approaches produced consistent results when applied within the same study population and to assess how estimates differed when external data sources were used.

### 2.2 Statistical Methods

#### 2.2.1 Cohort Follow‐Up Approach

The cohort follow‐up approach is considered the gold standard in determining HIV incidence [24], where HIV‐negative individuals are prospectively followed with repeated HIV testing. Incidence is calculated as the number of new HIV acquisitions per person‐year (PY) of follow‐up, with a 95% asymptotic confidence interval derived using the normal approximation on the log‐transformed incidence rate.

#### 2.2.2 Recency Testing Approach

The recency testing approach estimates HIV incidence using a cross‐sectional sample that includes both HIV acquisition status and results from a RITA. We applied the estimator proposed by Kassanjee et al. [25], with the 95% asymptotic confidence intervals calculated using the normal approximation on the log‐transformed incidence [26]. This estimator depends on the mean duration of recent infection (MDRI), defined as the average number of days classified as “recent,” and the false‐recent rate (FRR), the probability of misclassifying long‐term infections as recent.

We adopted a RITA that defines recent infection as limiting antigen avidity (LAg‐Avidity) normalized optical density (OD‐n) ≤1.5 with viral load > 1000 copies/mL [27]. For this RITA, the MDRI was 202 days (95% CI: 152, 260) and the FRR was 0% in Lima, Peru (subtype B). The MDRI estimate was derived by applying a 15% increase to the reported MDRI of 176 days (95% CI: 132, 226) for a Sedia‐based RITA [11], reflecting a longer MDRI observed with a Maxim‐based algorithm [27, 28]. Acute HIV infections (HIV RNA positive but HIV seronegative) were treated as HIV‐negative in the primary analysis. We also modified the Kassanjee estimator to account for missing recency testing results among some individuals who tested HIV‐positive (see Appendix A1). Sensitivity analyses varied the viral load threshold (>75 copies/mL) and the handling of acute HIV infections (as HIV‐positive or excluding them from the analysis).

#### 2.2.3 RG Approach

The RG approach, proposed by Mullick and Murray [13], leverages the association between RG and HIV incidences among MSM not using PrEP, based on historical data. The predicted HIV incidence in the absence of interventions is estimated by

$$\;{\hat{\lambda }}_{\mathrm{HIV},\mathrm{rG}}=1.8168+0.2218{\hat{\lambda }}_{\mathrm{rG}},$$

where ${\hat{\lambda }}_{\mathrm{rG}}$ is the estimated RG incidence.

For the primary analysis, we calculate ${\hat{\lambda }}_{\mathrm{rG}}$ as the total number of RG cases (at enrolment or during follow‐up) divided by the total PY of follow‐up, with a 95% asymptotic confidence interval based on the normal approximation of the log‐transformed incidence rate. To avoid double‐counting, repeated positive RG results within 6 weeks were counted as a single incident case, as RG may remain detectable for several weeks following acquisition and treatment. As a sensitivity analysis, we also estimate RG incidence using the first incident RG case during follow‐up, excluding prevalent cases at enrolment.

#### 2.2.4 Propensity Score Approach to Address Population Heterogeneity

Although the studies overlapped in time periods and geographic areas, participants’ characteristics differed. To improve counterfactual incidence estimates, we accounted for population heterogeneity using a propensity score (PS) weighting approach.

For each individual, the PS was defined as the probability of belonging to the target population given their observed covariates. PSs were estimated using logistic regression models with shared covariates and selected interaction terms. Balance after weighting was assessed using chi‐squared tests comparing marginal covariate distributions (Appendix A2). Individuals were then weighted to align the covariate distribution of the external population with that of the target population; a related but distinct PS weighting method was used for the recency‐testing approach [12].

#### 2.2.5 Comparison of Counterfactual Incidence Estimates

We specified the AMP (randomized clinical trial) and Sabes step 2 (observational cohort) populations as the target “gold standard” populations and estimated counterfactual incidence based on different approaches. Both studies provided prospective HIV follow‐up data, either without intervention or with interventions yielding incidence rates similar to placebo. However, participant characteristics likely differ due to varying eligibility criteria and the distinct profiles of individuals willing to enrol in clinical trials versus observational cohorts.

For each target population, the cohort follow‐up estimate served as the gold standard. We evaluated the performance of the recency‐testing and RG approaches when applied to data from the same study and assessed the performance of all three approaches when applied to data from external studies. In the latter case, we applied statistical adjustments to account for population differences and generate counterfactual incidence estimates aligned with the target population.

We anticipated closer agreement between estimation methods applied within the same study population than between estimates derived from different studies. Comparisons across studies were expected to reflect not only methodological differences, but also differences in calendar period, participant characteristics and underlying HIV risk profiles.

## 3 Results

### 3.1 HIV Incidence Estimates Based on Data Collected From Each Study

**FIGURE 1:**
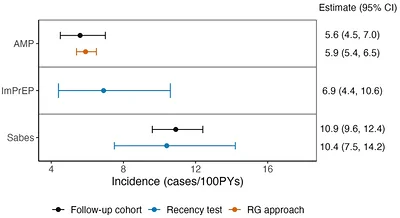
Estimated HIV incidence across studies using different approaches. Points represent estimated HIV incidence rates and horizontal bars represent 95% confidence intervals (CIs). Black points indicate prospective cohort follow‐up estimates, blue points indicate recency testing estimates and orange points indicate rectal gonorrhoea (RG)‐based estimates. Incidence is expressed as cases per 100 person‐years (PYs). AMP, antibody‐mediated prevention trial; ImPrEP, ImPrEP seroincidence study; Sabes, sabes study.

We first estimated HIV incidence for the AMP, ImPrEP seroincidence and Sabes studies using data collected directly from each study (Figure 1). In the AMP study, the estimated HIV incidence using the cohort follow‐up approach was 5.6 cases/100 PY (95% CI: 4.5, 7.0). Using the RG approach, 198 RG cases were identified over 1072.2 follow‐up PYs, yielding an HIV incidence estimate of 5.9 cases/100 PY (95% CI: 5.4, 6.5). In the ImPrEP seroincidence study, 33 recent HIV infections were identified by the RITA, resulting in an HIV incidence estimate of 6.9 cases/100 PY (95% CI: 4.4, 10.6). In the Sabes study, the cohort follow‐up approach yielded an incidence estimate of 10.9 cases/100 PY (95% CI: 9.6, 12.4). Using the recency testing approach, 139 recent HIV infections were identified by the RITA, resulting in an incidence estimate of 10.4 cases/100 PY (95% CI: 7.5, 14.2).

Overall, incidence estimates within each study were generally consistent across methods. The recency testing approach produced wider confidence intervals compared to the cohort follow‐up and RG approaches. Sensitivity analyses of the recency testing and RG approaches yielded similar estimates and are not shown here.

### 3.2 Estimates Based on Data Collected From Other Populations

#### 3.2.1 Estimates for a Clinical Trial (AMP Study) Population

We applied the PS weighting methods in Section 2.2.4 to provide incidence estimates for the AMP study using data collected from the Sabes and ImPrEP seroincidence studies. The covariates included in the PS model and their balance diagnostics are summarized in Table S1, which reports *p*‐values comparing covariate distributions before and after weighting.  After PS adjustment, the weighted marginal distributions of most covariates closely matched those observed in AMP.

**FIGURE 2:**
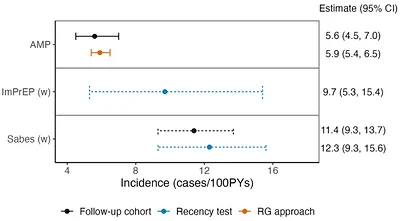
HIV incidence estimates for the AMP population using data from different studies and estimation approaches. Points represent estimated HIV incidence rates and horizontal bars represent 95% confidence intervals (CIs). Black points indicate prospective cohort follow‐up estimates, blue points indicate recency testing estimates and orange points indicate rectal gonorrhoea (RG)‐based estimates. Incidence is expressed as cases per 100 person‐years (PYs). AMP, antibody‐mediated prevention trial; ImPrEP, ImPrEP seroincidence study; Sabes, sabes study.

Figure 2 summarizes the estimated HIV incidences for the AMP population. Using the recency testing approach applied to the ImPrEP seroincidence data, the estimated HIV incidence was 9.7 cases/100 PY (95% CI: 5.3, 15.4). Using the Sabes study data, the incidence estimates were 11.4 (95% CI: 9.3, 13.7) and 12.3 (95% CI: 9.3, 15.6) cases/100 PY based on the follow‐up cohort and recency testing approaches, respectively.

Although PS weighting was used to adjust for covariate differences, the incidence estimates using both the ImPrEP seroincidence and Sabes data remained higher than the gold‐standard follow‐up estimate from AMP. Notably, the PS‐adjusted estimates from the ImPrEP and Sabes studies were higher than the corresponding unadjusted estimates shown in Figure 1. While the confidence interval for the adjusted ImPrEP estimate is wide and includes the AMP point estimate, the confidence intervals for the adjusted Sabes estimates do not overlap with the AMP estimate, suggesting a persistent discrepancy.

#### 3.2.2 Estimates for an Observational Cohort (Sabes Step 2) Population

We also applied the PS weighting methods to estimate incidence for the Sabes step 2 population, as an example for an observational cohort, using data from AMP and ImPrEP. Covariates and balance diagnostics are shown in Table S2. After weighting, most covariate distributions closely matched those in Sabes.

**FIGURE 3:**
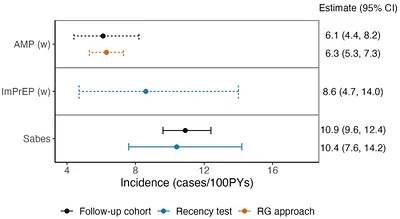
HIV incidence estimates for the Sabes step 2 population using data from different studies and estimation approaches. Points represent estimated HIV incidence rates and horizontal bars represent 95% confidence intervals (CIs). Black points indicate prospective cohort follow‐up estimates, blue points indicate recency testing estimates and orange points indicate rectal gonorrhoea (RG)‐based estimates. Incidence is expressed as cases per 100 person‐years (PYs). AMP, antibody‐mediated prevention trial; ImPrEP, ImPrEP seroincidence study; Sabes, Sabes study.

Using data from the AMP study, the estimated HIV incidence was 6.1 (95% CI: 4.3, 8.2) and 6.3 (95% CI: 5.3, 7.3) cases/100 PY using the follow‐up cohort and RG approaches, respectively (Figure 3). With data from the ImPrEP seroincidence study, the incidence estimate was 8.6 (95% CI: 4.7, 14.0) cases/100 PY based on the recency testing approach.

After applying PS weighting, the adjusted estimates from the AMP and ImPrEP studies were higher than the corresponding unadjusted estimates shown in Figure 1. However, the AMP‐based estimates remained substantially lower than the gold‐standard cohort follow‐up estimate from the Sabes study, with non‐overlapping confidence intervals. In contrast, the confidence interval for the adjusted ImPrEP estimate was wide and included the gold‐standard Sabes estimate.

## 4 Discussion

When applied to data collected within the same study, all three approaches—cohort follow‐up, recency testing and RG—produced consistent HIV incidence estimates. Specifically, cohort and RG‐based estimates were closely aligned in the AMP study, while cohort and recency‐based estimates agreed in the Sabes study. These findings support the internal validity of each method and demonstrate their utility when applied in a consistent population and context.

However, when these methods were used to estimate incidence for an external target population, substantial discrepancies emerged—even after adjusting for observed covariate differences via PS weighting. For the AMP clinical trial population, PS‐adjusted incidence estimates based on Sabes and ImPrEP data ranged from 9.7 to 12.3 cases per 100 PY, well above AMP's gold‐standard follow‐up estimate. In contrast, for the Sabes population, adjusted estimates based on AMP and ImPrEP data ranged from 6.1 to 8.6 cases per 100 PY—lower than the gold‐standard Sabes cohort follow‐up estimate. These differences highlight the challenges of using external data to estimate counterfactual incidence.

PrEP use is unlikely to explain those discrepancies. Although AMP participants had access to oral PrEP, actual use was limited: the average PrEP use among participants in the four Lima sites was 8.2% for detectable TFV‐DP in dried blood spot and 4.3% for TFV‐DP > 700 fmol/punch, a protective level consistent with 4 or more doses/week. We also derived an enhanced incidence estimate that excluded follow‐up time during self‐reported PrEP use (details in Appendix A3), yielding 5.8 (95% CI: 4.5, 7.1) cases per 100 PY—nearly identical to the unadjusted estimate. This suggests that the low levels of PrEP use in AMP had minimal impact on overall incidence estimation.

Temporal trends are another potential source of bias but likely played a limited role. While the Sabes study was conducted earlier and the ImPrEP study more recently, national HIV surveillance data indicate that HIV incidence in Peru has remained relatively stable in the past 10 years [29, 30]. In addition, PrEP uptake in Peru has historically remained relatively low outside of research and demonstration projects, particularly during much of the time covered by these studies [31]. The limited population‐level coverage of PrEP may help explain the absence of a strong calendar time trend in HIV incidence across studies. Although the earlier timing of Sabes—before PrEP availability—may partly explain its higher incidence, time alone does not fully account for the observed differences across studies.

A more plausible explanation is residual unmeasured confounding and population heterogeneity. Despite PS adjustment, substantial differences likely remain between populations in clinical trials (such as AMP) versus observational cohorts (such as Sabes and ImPrEP). Clinical trial participants may represent a lower‐risk subgroup due to selective enrolment criteria or self‐selection bias. This is consistent with patterns observed in other trials—for instance, the recently completed MOSAICO study [32]. In Peru, the HIV‐1 incidence rates in the vaccine and placebo groups were 4.74 per 100 PY (95% CI: 3.22–6.72) and 6.44 per 100 PY (95% CI: 4.60–8.76), respectively. These findings underscore the importance of understanding and fully accounting for heterogeneity in study populations when combining different studies to generate counterfactual incidence estimates.

In addition, the study populations also differed in several other important ways, including recruitment strategies, eligibility criteria, prevention access, healthcare engagement and underlying behavioural risk profiles. For example, the AMP study enrolled participants willing to participate in a randomized clinical trial with frequent study visits and prevention services, whereas the Sabes and ImPrEP studies recruited participants through different community‐based and public health settings. These differences may influence both measured and unmeasured HIV risk factors and may not be fully addressed through PS adjustment.

More comparable counterfactual incidence estimates may be achievable in settings where external studies enrol populations with similar eligibility criteria, recruitment mechanisms, calendar periods and prevention environments. Statistical adjustment approaches such as PS weighting are likely to perform better when the underlying populations are more exchangeable and when key behavioural and biologic risk factors are measured consistently across studies.

Despite limitations in generalizability, the RG approach closely aligned with the gold‐standard follow‐up cohort estimates in the AMP population, supporting its use as a proxy measure under certain conditions. However, it depends on the assumption that the ecological association between RG and HIV incidence observed in prior studies is transportable across populations and time, which may not hold given differences in HIV epidemiology, testing practices, treatment coverage and uptake of prevention interventions. For example, a recent study conducted in sexual health clinics for MSM in England found a divergence in HIV and RG trends from 2011 to 2018, likely due to expanded HIV testing, prompt Antiretroviral therapy initiation and increased viral suppression in persons living with HIV [33]. Moreover, fully validating the RG−HIV relationship within a local setting would itself require reliable HIV incidence data, which may substantially reduce the practical need for the RG approach. Instead, the plausibility of RG‐based estimates may need to be assessed indirectly through triangulation with other data sources, temporal consistency and comparison with recency testing or cohort‐based estimates. Overall, the RG approach should be interpreted cautiously as its validity depends on the transportability of the underlying RG−HIV association.

Similarly, the recency testing approach performed well when using the internal data, producing estimates consistent with cohort‐based incidence. This aligns with the successful application of recency testing in the PURPOSE trials and supports its use in settings where prospective follow‐up is not feasible. Still, the method's reliability depends on accurate calibration of the recency assay, appropriate case definitions and access to recent testing data.

Taken together, our results support the value of multiple counterfactual estimation strategies—especially when used in tandem. When applied within the same study, recency testing and RG methods can serve as practical alternatives to cohort follow‐up. When using external data, PS adjustment improves comparability but may not fully account for underlying differences in risk. Specifically, our findings demonstrate that estimates derived from external populations may systematically overestimate or underestimate the true incidence in the target population because of residual heterogeneity and selection effects. Therefore, counterfactual estimates should not be interpreted as definitive benchmarks for efficacy on their own. Instead, triangulation across multiple methods and external data sources may help define a plausible range of background incidence and provide supportive evidence when interpreted together with study design, population characteristics and other efficacy data.

Finally, we note that our approach is constrained by the availability of specific data types and studies. While Lima presented a unique setting where multiple relevant datasets could be harmonized and compared, such opportunities are rare. In other settings, access to reliable recency testing, RG surveillance or detailed cohort follow‐up data may be limited. This highlights the need for ongoing investment in surveillance infrastructure and standardized data collection to support future counterfactual incidence estimation efforts.

## 5 Conclusions

Counterfactual HIV incidence estimates can support evaluation of new PrEP agents when direct placebo comparisons are not possible. Estimates from recency‐testing and RG approaches are promising when prospective follow‐up is infeasible, but their validity depends on data quality and population similarity. PS methods improve comparability across populations but cannot fully account for unobserved differences. Triangulating across methods and sources can improve confidence in counterfactual estimates, which should be interpreted with careful attention to population context.

## Author Contributions

JFR and AD conceptualized the study; AD supervised the study and acquired funding. Resources (data and laboratory results) were provided by RA, TST, JS, JRL, VGV, RC, BG, CFC and JFR. Project administration was by DP‐S and JOA. FG, SD, QW, RA and TST contributed to the methodology used (incidence estimation through analysis of longitudinal cohort data, LAg avidity testing and incidence of rectal gonorrhoea). RA, DP‐S, TST and JS conducted or facilitated investigations (mainly conduct of laboratory assays of limiting antigen avidity). The formal analyses were conducted by FG and SD, with data curation by SP. FG, QW and AD wrote the original draft and all authors reviewed and approved the final version.

## Conflicts of Interest

JR is an employee of Gilead Sciences Inc., the main funder of this analysis. The funder had no role in study design, data collection, analysis, interpretation or the decision to publish. All other authors declare that they have no conflicts of interest.

## Supporting information




**File S1**: jia270204‐sup‐0001‐Table S1‐S2.docx

## Data Availability

The data that support the findings of this study are available on request from the corresponding author. The data are not publicly available due to privacy or ethical restrictions.

## APPENDIX A Details on Statistical Methods

### A1 Modified Kassanjee et al. (2012) Estimator

Let $T^{*}$ denote a cutoff time, such that a recent HIV infection is defined as an acquisition occurring within the past $T^{*}$ time units. The incidence estimator of the recency testing approach adopts two parameters that characterize the recency test based on $T^{*}$: mean duration of infection (MDRI) $\Omega_{T^{*}}$ and false‐recent rate (FRR) $\beta_{T^{*}}$.

For individual i=1,…,N in the cross‐sectional sample, let $D_{i}$ be the indicator for HIV test status, where $D_{i}=1$ indicates an HIV‐positive result; $R_{i}$ denote the indicator of recent HIV infection classification by the recency test; and $Q_{i}$ denote the indicator of whether the recency test was administered.

Then, the HIV incidence can be estimated by

$$\;{\hat{\lambda }}_{H\mathrm{IV},\mathrm{recency}}=\frac{\sum_{i=1}^{N}D_{i}Q_{i}\left(R_{i}-{\hat{\beta }}_{T^{*}}\right)}{\sum_{i=1}^{N}\left(1-D_{i}\right)\left({\hat{\Omega }}_{T^{*}}-{\hat{\beta }}_{T^{*}}T^{*}\right)}\;\times \frac{\sum_{i\;=1}^{N}D_{i}}{\sum_{i\;=1}^{N}D_{i}Q_{i}}.$$

### A2 PS Weighting

We pool data from both the source population (with follow‐up data) and the target population (for which we aim to estimate incidence). For individual i=1,…,N in the pooled population, let $X_{i}$ be the vector of covariates and $S_{i}$ be the indicator of population membership, where $S_{i}=0$ if individual i belongs to the source population and $S_{i}=1$ if from the target population. The PS for individual i is defined as

$$\;\omega_{i}=P\left(\;S_{i}=1|X_{i}\right),$$

and is estimated by fitting a logistic regression model to the pooled data.

Let $\delta_{i}$ be the indicator of HIV acquisition during follow‐up and $\tau_{i}$ the follow‐up time for individual i in the source population (i.e. with $S_{i}=0$). Then, the PS‐adjusted estimator for the HIV incidence in the target population is given by

$$\;{\hat{\lambda }}_{HIV,fu}^{PS}=\frac{\sum_{i=1}^{N}\frac{{\hat{\omega }}_{i}}{1-{\hat{\omega }}_{i}}\left(1-S_{i}\right)\delta_{i}\;}{\sum_{i=1}^{N}\frac{{\hat{\omega }}_{i}}{1-{\hat{\omega }}_{i}}\left(1-S_{i}\right)\tau_{i}}\;,$$

where ${\hat{\omega }}_{i}$ is the estimated PS for individual i.

For the RG approach, the RG incidence in the target population can be similarly estimated, and one can obtain the estimate for the counterfactual HIV incidence in the target population using the linear relationship in Section 2.2.3.

To account for population heterogeneity between the AMP and Sabes populations, we include the main effect for the covariates in the PS model. This specification achieves good covariate balance between the target population and the PS‐weighted source population, as demonstrated in Tables S1 and S2. After weighting, the *p*‐values from the Wald tests for nearly all covariates exceed 0.05 after weighting (treating PS weights as fixed in the hypothesis testing), indicating adequate balance.

When leveraging data from the ImPrEP seroincidence study, we further include interaction terms in the PS model to improve PS model fit. Interaction terms are selected using a forward selection procedure, in which candidate interaction terms are sequentially added based on statistical significance, and the final model is chosen to minimize the Akaike information criterion. After PS adjustment, Wald test *p*‐values for nearly all covariates increase relative to their unadjusted values, indicating improved covariate balance.

### A3 Enhanced Incidence Estimator Accounting for PrEP Use

For individual i=1,…,N in the population, let $\tau_{i}$ be the follow‐up time of individual i. Additionally, let $\tau_{i}^{*}$ and $\delta_{i}^{*}$ be the no‐PrEP follow‐up time and the HIV acquisition indicator during no‐PrEP follow‐up, respectively. Then, to form a no‐PrEP cohort that has a similar profile as the original cohort, we weight subject i by $\;w_{i}=\tau_{i}/\tau_{i}^{*}$ and calculate a no‐PrEP incidence as

$$\;{\tilde{\lambda }}_{fu}=\frac{\sum_{i=1}^{N}w_{i}\delta_{i}^{*}}{\sum_{i=1}^{N}w_{i}\tau_{i}^{*}}\;\;=\frac{\sum_{i=1}^{N}w_{i}\delta_{i}^{*}}{\sum_{i=1}^{N}\tau_{i}}.$$

In this calculation, we remove the individuals who have 0 no‐PrEP follow‐up time, which are those using PrEP during the entire follow‐up time.

After excluding follow‐up time during self‐reported PrEP use, the total follow‐up time is 1386.6 PYs, and the incidence estimate is 5.8 (95% CI: 4.5, 7.1) cases per 100 PY.
