# Supplementary material for: Comparing Approaches for Estimating Counterfactual HIV Incidence Among Populations With High Vulnerability to HIV in Lima, Peru: A Multi‐Study Comparative Analysis
Source: J Int AIDS Soc. 2026 Sep 15;29(9):e70204. doi: 10.1002/jia2.70204 (PMC13575769; doi:10.1002/jia2.70204)
Supplement: Supplementary file 1 [file JIA2-29-e70204-s001.docx]

**Supplementary Material**

Table S1: p-values* for covariate distributions before and after PS adjustment with AMP study (clinical trial) as the target population.

| Covariates | AMP vs Sabes step 2 | | AMP vs Sabes step 1 | | AMP vs ImPrEP  seroincidence | |
| --- | --- | --- | --- | --- | --- | --- |
| Before/After Adjustment | Before | After | Before | After | Before | After |
| Age at Enrollment | 0.127 | 0.703 | 0.039 | 0.941 | <0.001 | 0.480 |
| Sexuality** | <0.001 | 0.787 | <0.001 | 0.748 | <0.001 | 0.709 |
| Sexual Role | <0.001 | 0.630 | <0.001 | 0.840 | <0.001 | 0.002 |
| Post Secondary Education | 0.024 | 0.749 | 0.349 | 0.903 | - | - |
| Complete Secondary Education | - | - | - | - | 0.319 | 0.609 |
| Below Minimum Wage | 0.016 | 0.600 | 0.052 | 0.381 | <0.001 | 0.059 |
| # Sex Partner | <0.001 | 0.993 | <0.001 | 0.992 | - | - |
| # Sex Partner (Coerced) | - | - | - | - | 0.658 | 0.33 |
| Anal Sex without Condom | <0.001 | 0.931 | <0.001 | 0.941 | <0.001 | 0.014 |
| Reported Sex with Partner living with HIV | 0.006 | 0.878 | 0.004 | 0.882 | 0.124 | 0.162 |
| Have Main Partner | <0.001 | 0.878 | <0.001 | 0.932 | - | - |
| Always use Condom with Main Partner | <0.001 | 0.946 | <0.001 | 0.778 | - | - |
| Buy or Sell Sex | <0.001 | 0.889 | <0.001 | 0.820 | - | - |
| Binge Drinking | 0.008 | 0.843 | 0.007 | 0.960 | <0.001 | 0.088 |
| Sex Under Influence | <0.001 | 0.778 | <0.001 | 0.714 | - | - |

* p-values that are from the Pearson chi-square test, where a smaller p-value indicates more evidence against the null hypothesis that a covariate has the same distribution in two populations.

** The variable “Sexuality” describes the sexual orientation and gender for the participants, with categories bisexual, heterosexual, homosexual, and trans.

Abbreviations: AMP, Antibody-Mediated Prevention trial; PS, propensity score; HIV, human immunodeficiency virus; ImPrEP, Implementation PrEP; TG, transgender persons; MSM, men who have sex with men.

Table S2: p-values* for covariate distributions before and after PS adjustment with Sabes step 2 study (observational cohort) as the target population.

| Covariates | Sabes step 2 vs AMP | | Sabes step 2 vs ImPrEP seroincidence | |
| --- | --- | --- | --- | --- |
| Before/After Adjustment | Before | After | Before | After |
| Age at Enrollment | 0.127 | 0.717 | <0.001 | 0.158 |
| Sexuality ** | <0.001 | 0.899 | <0.001 | 0.082 |
| Sexual Role | <0.001 | 0.016 | <0.001 | 0.163 |
| Post Secondary Education | 0.024 | 0.519 | - | - |
| Complete Secondary Education | - | - | <0.001 | 0.027 |
| Below Minimum Wage | 0.018 | 0.944 | <0.001 | 0.023 |
| # Sex Partner | <0.001 | 0.408 | - |  |
| # Sex Partner (Coerced) | - | - | <0.001 | 0.001 |
| Anal Sex without Condom | <0.001 | 0.902 | 0.105 | 0.485 |
| Reported Sex with Partner living with HIV | <0.001 | 0.512 | 0.016 | 0.658 |
| Have Main Partner | <0.001 | 0.602 | - |  |
| Always use Condom with Main Partner | <0.001 | 0.665 | - |  |
| Buy or Sell Sex | <0.001 | 0.679 | - |  |
| Sell Sex | - | - | 0.367 | 0.316 |
| Binge Drinking | 0.009 | 0.535 | <0.001 | 0.073 |
| Sex Under Influence | <0.001 | 0.894 | - |  |

* p-values are from the Pearson chi-square test, where a smaller p-value indicates more evidence against the null hypothesis that a covariate has the same distribution in two populations.

** The variable “Sexuality” describes the sexual orientation and gender for the participants, with categories bisexual, heterosexual, homosexual, and trans.

Abbreviations: AMP, Antibody-Mediated Prevention trial; PS, propensity score; HIV, human immunodeficiency virus; ImPrEP, Implementation PrEP; TG, transgender persons; MSM, men who have sex with men.
